# 

**Published:** 1912-02-10

**Authors:** 


### Sulphate of Soda.

Sulphate of soda is one of the most admirable medicines we possess and deserves to be more popular than it is. I have constantly ordered it, with or without a small addition of sulphate of magnesia, for the out-patients of the hospital, and regard it as the best substitute for Carlsbad and other natural mineral waters.—*Sir H. Thompson.*

### Gifts.

Mr. Wm. Joshua Smith, of Brighton, has left £100, at his wife's death, to the Sussex County Hospital.

Mr. John Jackson Shiers, of Cheshire and Oldham, has left £500 to the Oldham Infirmary.

Mr. Francis Tagart, of Bristol, has left £1,000 to the Bristol Royal Infirmary.

Miss Marianne Starmer, of Wood Green, has left £350 to the Wood Green Hospital.

The West Norfolk and Lynn has received £50 from H.M. Queen Alexandra.

Mr. William Medcalf, of Highbury, has left £200 each to the Metropolitan Hospital, the London Hospital, the Queen's Hospital for Children, Hackney Road, and the City of London Hospital for Diseases of the Chest.

Miss Margaret Thorpe, of Knightsbridge, W., has bequeathed the ultimate residue of her estate to be equally divided between St. George's Hospital and the Cancer Hospital, Fulham Road, probably about £3,000 to each

Through the kindness of the Duchess of Westminster, who gave a theatrical entertainment at Eaton Hall on behalf of the Chester hospitals and Grosvenor Hospital for Women, Mr. W. Davidson, the secretary, has received £100 as a donation towards its funds.

### Buildings Contemplated and in Progress.

Bolton.—Designs have been submitted by architects, and are now being adjudged by the assessor, Mr. John B. Gass, F.R.I.B.A., for a new nurses' home at the Bolton Infirmary. The competition is confined to principals practising within the Borough, and the building is to be known as an Edward VII. memorial.

Christchurch.—At a cost of £2,200, additions are being carried out at the Christchurch Infirmary. Mr. C. W. Ball, of Southsea, is the architect.

Carnarvon.—Mr. R. L. Jones, M.S.A., has prepared the plans, etc., for the proposed new infirmary at Carnarvon for the union, and tenders are being considered.

East Barnet.—The Joint Isolation Hospital Committee have appointed a sub-committee to go into the question of alterations at their hospital, including the erection of an observation block.

Garstang.—An isolation hospital is being built at a cost of £1,457, from a design prepared by the Rural Council's Surveyor, Mr. J. Cook.

Greenock.—A design for new baths and washhouses has been prepared by the Master of Works, Mr. J. Low. The cost is estimated to be £5,000.

Honiton.—Extensive building works are projected at the workhouse, Honiton, including the erection of new casual wards. Mr. A. T. Redfern is the architect, and tenders have been submitted for the several works.

Limerick.—Mr. W. Rosengrave is architect for the proposed additions to the Infirmary at Limerick Workhouse; the estimated cost is £2,500.

London.—Certain alterations are proposed at the St. James' Infirmary, Wandsworth, on the Needle Room block of buildings. Tenders are now being considered.

### The Week's Novelties.

### THE KEROL PREPARATIONS.

All readers must have been struck by the page in our business columns last week devoted to the Kerol preparations, chiefly to the Kerol disinfecting-powders. The art of disinfecting is still a kind of black magic to the lay public, which makes no effort to discriminate between one preparation and another. Hospitals are not so careless, but for that reason must scrutinise carefully every claim. Kerol, for example, is stated to be at least ten times more powerful than a 15-per-cent. pure carbolic-acid powder. Outside opinion has described it as practically non-poisonous. Unlike perchloride of mercury, we learn, Kerol can be used in conjunction with soap. The upshot is that no institutional officer can afford not to try it. Many have come to do so through the other well-known Kerol preparations—the Kerol toilet-soap and the toilet Lano-Kerol, an antiseptic cream which the present writer has tested most successfully for incipient herpes and the rough lips that the east wind leaves behind it. The manufacturers to write to are Quibell Brothers, Ltd., 159 Castlegate, Newark.

### BOOKS RECEIVED.

John Murray.—"Further Researches into Induced Cell-Reproduction and Cancer," by H. C. Ross; "The Anopheline Mosquito," by R. C. Dauglish.

Cassell and Co.—"British Red Cross Society Training Manual," by James Cantlie, M.A., M.B.; "Essays on Duty and Discipline"; "A System of Surgery," by C. C. Choyce, M.D.

Wells Gardner, Darton and Co., Ltd.—"The Story of Sintram and His Companions," by M. Macleod; "Marvels of Man's Making," by J. Lea.

E. and S. Livingstone.—"The Care of Infants and Young Children," by A. Dingwall Fordyce, M.D.

### Florence Nightingale Memorial.

THE following contributions have been received by Mr. G. Q. Roberts. They are for a statue unless otherwise noted :—

Anonymous (for the Annuities Fund), £1,000; Worshipful Company of Merchant Taylors, £21; collected by Matron, British Hospital, Buenos Ayres, £12 13s. 2d.; J. G. Griffiths, Esq., £10 10s.; Joseph Howard, Esq., J.P., £10 10s.; Lady Octavia Shaw Stewart, £10 10s.; V. W. B. Van De Weyer, Esq., J.P., £10; W. H. A. Wharton, Esq., £10; collected by Matron, General Hospital, Nottingham, £7 12s. 6d.; collected by Matron, General Hospital, Nottingham (annuities), £2 12s. 6d.; collection by the Mayor of Swansea (1911), £6 14s. 6d.; the Misses Amy, Kate, and Rose Neville, £6; Sir William Wedderburn, Bart., £5 5s.; Colonel C. Swan, £5; Lord Zetland, £5; Sir Robert Usher, Bart., £5; Messrs. Robson and Ross, £5; Robert Hermon Hodge, Esq., £5; William Wyndham, Esq., £5; H. E. Monro, Esq., £5; Officers of the 1st Life Guards, £5; Sir George Macalpine, Knt., £5; the Ven. Canon T. H. A. Houblon, D.D., £5; Rev. Canon F. Warre £3; "M. W." (annuities), £3; Sir Robert Mowbray, Bart., £2 2s.; Mrs. Henry Wigram, £2 2s.; Sir Harry Verney, Bart., £2 2s.; Dr. Anthony Traill, £2 2s.; the Dowager Countess of Lytton, £2 2s.; Mr. and Mrs. J. H. Walter, £2 2s.; Mrs. Wood, £2 2s.; Field-Marshal Sir George White, £2 2s.; the Very Rev. Dr. Henry Wace, £2 2s.; Colonel Sir Fitzroy Donald Maclean, £2 2s.; Miss Macnabb, £2 2s.; Rev. J. C. Hill, £2 2s.; Lieut.-Gen. Sir Josceline Wodehouse, K.C.B., £2; Lieut.-Gen. Hon. Sir Frederick Stopford, K.C.M.G., £2; Sir Oliver Lodge, Knt., £2; Officers, N.C.O.'s, and Men, 53rd Co. R.G.A., £1 10s.; Colonel Morey Quayle-Jones, C.B., £1 1s.; General the Hon. Sir Neville Lyttelton, G.C.B., £1 1s.; Mrs. Alfred Huth, £1 1s.; Dr. W. H. Willcox, £1 1s.; Sir Henry Waterfield, G.C.I.E., K.C.S.I., £1 1s.; Canon Wardell-Yerburgh, £1 1s.; Maud Lady Wood, £1 1s.; General Sir George Luck, G.C.B., £1 1s.; Major Sir Frederick Wodehouse, K.C.V.O., £1 1s.; General J. N. Young, £1 1s.; Sir Mark Stewart, £1 1s.; Miss F. C. Tubbs, £1 1s.; Lady Flower, £1 1s.; Captain L. P. Walsh, £1 1s.; Sir Henry Wilson, K.C.M.G., £1 1s.; Colonel and Mrs. R. H. Jelf, £1 1s.; J. W. Lovibond, Esq., J.P., £1 1s.; A. A. Lloyd, Esq., £1 1s.; Surgeon-General H. R. Whitehead, C.B., £1 1s.; L. C. Hopkins, Esq., £1 1s.; Walter Scrymgeour, Esq., £1 1s.; Lieut.-Colonel C. Greenhill-Gardyne, £1 1s.; Sir James Lemon, Knt., £1 1s.; Mrs. Smith, £1 1s.; His Honour Judge G. H. Lea, £1 1s.; Sir Robert Hunter, Knt., £1 1s.; Major and Mrs. H. Hewetson, £1 1s.; Lord Mackenzie, £1 1s.; Vernon Lushington, Esq., Q.C., £1; Colonel H. Wood, C.B., £1; the Marquis of Linlithgow, £1; Sir Edmund Giles Loder, Bart., £1; Sir Thomas Lees, Bart., £1; Lady Strachey, £1; Miss Tatlock, £1; Hon. F. W. Lambton, £1; T. E. Yorke, Esq., £1; Captain de Hoghton, R.N., £1; Viscount Valentia, £1; the Very Rev. G. W. Kitchin, £1; A. Thorold, Esq. (annuities), £1; C. Turnor, Esq., £1; Surgeon Lieut.-Col. Sir Warren Crook-Lawless, Knt., £1; Mrs. Waddingham, £1; Queen's Nurses (additional), 10s. 6d.; Mrs. Harley, 10s.; Lady Thring, 10s.; Rev. Preb. I. G. Lloyd, 5s.; Major E. J. Lugard, D.S.O., 5s.; the Right Rev. Dr. A. M. Knight, 5s.; Miss L. F. Waring, 2s. 6d.; "A Friend," 2s. 6d.; Miss Courtauld, 1s.

